# Recruited and Tissue-Resident Natural Killer Cells in the Lung During Infection and Cancer

**DOI:** 10.3389/fimmu.2022.887503

**Published:** 2022-07-01

**Authors:** Miriam Franklin, Emma Connolly, Tracy Hussell

**Affiliations:** Lydia Becker Institute of Immunology and Inflammation, Manchester Collaborative Centre for Inflammation Research (MCCIR), University of Manchester, Manchester, United Kingdom

**Keywords:** natural killer (Nk) cell, tissue-resident natural killer cells, lung cancer, immune cell recruitment, chemokines, extracellular matrix, tissue-specific immunity

## Abstract

Natural killer (NK) cells are an important component of the innate immune system, and have a key role in host defense against infection and in tumor surveillance. Tumors and viruses employ remarkably similar strategies to avoid recognition and killing by NK cells and so much can be learnt by comparing NK cells in these disparate diseases. The lung is a unique tissue environment and immune cells in this organ, including NK cells, exist in a hypofunctional state to prevent activation against innocuous stimuli. Upon infection, rapid NK cell infiltration into the lung occurs, the amplitude of which is determined by the extent of inflammation and damage. Activated NK cells kill infected cells and produce pro-inflammatory cytokines and chemokines to recruit cells of the adaptive immune system. More recent evidence has shown that NK cells also play an additional role in resolution of inflammation. In lung cancer however, NK cell recruitment is impaired and those that are present have reduced functionality. The majority of lung NK cells are circulatory, however recently a small population of tissue-resident lung NK cells has been described. The specific role of this subset is yet to be determined, but they show similarity to resident memory T cell subsets. Whether resident or recruited, NK cells are important in the control of pulmonary infections, but equally, can drive excessive inflammation if not regulated. In this review we discuss how NK cells are recruited, controlled and retained in the specific environment of the lung in health and disease. Understanding these mechanisms in the context of infection may provide opportunities to promote NK cell recruitment and function in the lung tumor setting.

## Introduction

**Figure 1:**
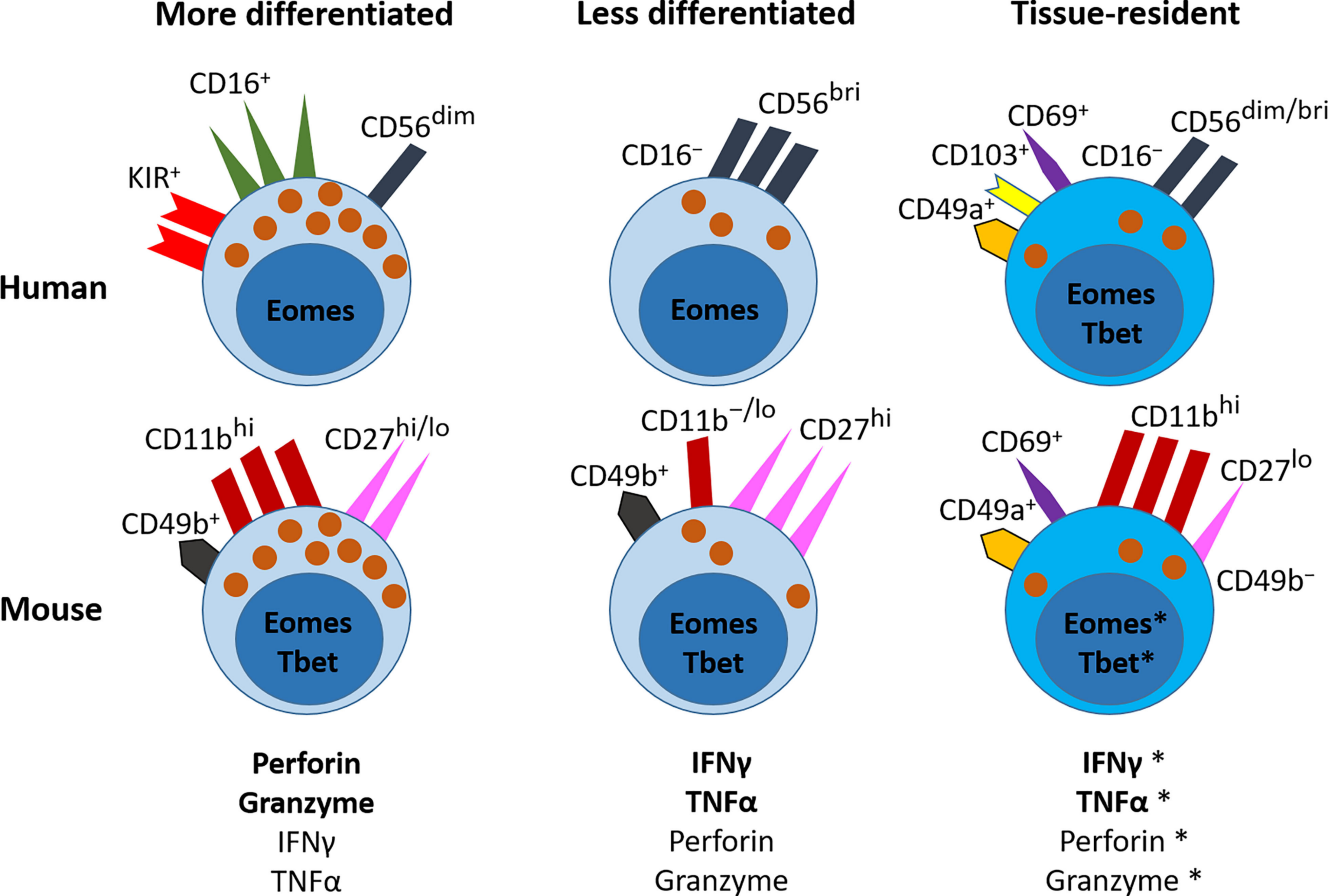
NK cells in the lung. NK cells are part of the ILC family, and in humans are classified into more or less differentiated on the basis of their expression of CD16. In mice, the relative levels of CD11b and CD27 correspond to their differentiation status. More differentiated NK cells predominantly express granules that contain perforin and granzyme, and to a lesser extent they produce the cytokines IFNγ and TNFα. In the human lung, they express CD16, CD56 and KIRs on their cell surface, and are controlled by the transcription factor Eomes. In the mouse lung, they express CD49b, high levels of CD11b, and variable levels of CD27, and are controlled by the transcription factors Tbet and Eomes. The less differentiated NK cells predominantly produce IFNγ and TNFα, but can also release perforin and granzymes. In the human lung, these NK cells do not express CD16 and express no/low levels of KIRs, but they express higher levels of CD56 than mature NK cells. They are also regulated by Eomes. In the mouse lung, these less differentiated NK cells express CD49b, no/low levels of CD11b, and high levels of CD27; and are regulated by Tbet and Eomes. In the human lung, tissue resident NK (trNK) cells express the cell-surface receptors CD69, CD49a and/or CD103; they are CD56^+^ but CD16^−^; they produce IFNγ and TNFα, and have low expression of lytic granules. They express both Eomes and T-bet. In the mouse lung, trNK cells express do not express CD49b, but express CD49a, CD69 and CD11b, and low levels of CD27. They are thought to express both Eomes and Tbet. Further detail on the cell surface markers that characterise these NK cell subsets in the human and mouse lung is shown in **Table 1**.*This is an area where further research is needed.

NK cells belong to the innate arm of the immune system, with roles analogous to those of CD8^+^ T cells of the adaptive immune system. They are part of the innate lymphoid cell (ILC) family, which also includes helper type 1, type 2 and type 3 ILCs (ILC1s, ILC2s and ILC3s). ILCs are categorized by the cytokines they express, which mirror those expressed by T helper (Th) cells Th1, Th2 and Th17 (1). NK cells share some similarity with ILC1s in that they both produce interferon-γ (IFNγ) and tumor necrosis factor-α (TNFα). However, NK cells, like CD8^+^ T cells, also produce granzyme B and perforin, which promote the lysis of target cells (1, 2) (**Figure 1**). Additionally, helper type ILCs are only found in tissues, whereas NK cells are a predominantly circulatory population (3, 4).

NK cells (and other ILCs) derive from common lymphoid progenitor cells in the bone marrow. After differentiation to NK cell precursors, they leave the bone marrow in an immature state and then proceed through several stages of maturation and differentiation in secondary lymphoid tissues, from where they exit to the peripheral blood (5, 6).

NK cells are important in the early stages of host defense, as they exist in a poised effector state (7). Their activation is strictly controlled by numerous activating receptors (for example, CD16, NKG2D, activating killer cell immunoglobulin-like receptors (KIRs) and NKp46) and inhibitory receptors (for example, inhibitory KIRs and NKG2A) that are expressed on their cell surface, and the balance of activating to inhibitory signals received by the NK cell determines its fate. CD16 (FcγRIII receptor) is the only receptor for which ligation on its own by antibody is sufficient to induce NK cell activation, leading to antibody-dependent cell cytotoxicity (ADCC). All other activating receptors require multiple interactions, which are counter-balanced by ligation of inhibitory receptors; for example, TIGIT and DNAM-1 bind to the same ligands and have opposing effects (8). NK cells also respond to soluble environmental cues such as pathogen-associated molecular patterns (PAMPs), damage-associated molecular patterns (DAMPs) and cytokines; in particular interleukins (IL) IL-2, IL-12, IL-18, IL-15 and type I IFNs which promote NK cell activation (9). Conversely, transforming growth factor-β (TGFβ) has many inhibitory effects on NK cells, which include reducing NK cell cytotoxicity and IFNγ production (9–11). These cytokine signals combine with the signals NK cells receive through their activating and inhibitory receptors to determine whether the NK cell activation threshold is reached (9).

Activating receptors bind to ligands associated with damage and stress (e.g. PAMPs and DAMPs), whereas inhibitory receptors bind to ‘self’ molecules — major histocompatibility complex class I (MHC-I), also known as human leukocyte antigen (HLA) — that are expressed on the surface of all nucleated host cells. In health, these interactions maintain NK cell inhibition. However, during infection, NK cells receive more activating receptor signals, which outweigh the inhibitory signals, thereby overcoming the threshold for NK cell activation. In a similar way, MHC-I molecules are often downregulated on the surface of tumor cells, reducing the amount of inhibitory signaling and enabling NK cell activation. This downregulation of MHC-I is known as the ‘missing self’ hypothesis (12). Once an NK cell is activated, either by antibody cross-linking of CD16 (ADCC) or by ligand binding to NK cell activating receptors, the interacting target cell is lysed. Lysis occurs *via* two different pathways: release of lytic granule content (perforin and granzyme), or death receptor signaling. In the first mechanism, the NK cell forms an immunological synapse with the target cell, and granules containing the lytic mediators perforin and granzyme B are released (13). Perforin creates pores in the target cell surface, through which granzyme B enters to induce apoptosis through cleavage and activation of caspase-3, as well as caspase-independent mechanisms (14). An alternative mechanism of NK cell killing is *via* death receptor signaling where NK cells expressing Fas ligand (FasL) or TRAIL, apoptosis-inducing members of the TNF family, bind their conjugate receptors Fas and TRAIL-R that are expressed on target cells. This receptor-binding interaction triggers the cleavage and activation of caspase-8, again leading to apoptosis of the target cell (15). The killing ability of NK cells is not limited to a single target cell — NK cells are able to sequentially kill multiple target cells, with *in vitro* studies showing NK cell serial killing of up to 10 target cells over a 6-hour period (16, 17).

As part of the innate immune system, the traditional view was that NK cells would not exhibit memory-like or adaptive-like properties. In fact innate immune cells, including NK cells, have been found to exhibit some form of immunological memory, also known as trained immunity (18). Various different memory-like NK cell subsets have been described in both humans and mice, including tissue-resident NK cells in the liver (19, 20), adaptive-like tissue-resident NK cells in the lung (21), pregnancy-trained decidual NK cells (22), and *in vitro* generated cytokine-induced memory-like NK cells (generated following IL-2/IL-15/IL-12/IL-18 stimulation) (23). These cells are generally characterized by NKG2C expression, and show increased cytokine production, cytotoxicity and proliferative capacity upon re-challenge (19, 21–24).

This review will focus on what is known about the specific phenotype of NK cells in the lung in both humans and mice, and how the NK cell population in this organ is affected in different disease states. In particular, this review will compare and contrast the mechanisms regulating NK cell function and recruitment in the settings of infection and cancer. Understanding these mechanisms will help identify new ways to promote NK cell recruitment to lung tumors, which may be applicable in a clinical setting.

### Comparison of NK Cell Subtypes in the Blood and in the Lung

In healthy human blood and lungs, NK cells make up around 10% of the population of lymphocytes (25, 26). NK cells are categorized based on the level of expression of CD56 (bright (br) and dim) and CD16 and include two broad subsets: CD56^br/dim^CD16^−^ and CD56^dim^ CD16^+^ (27). We will refer to these as CD16^−^ and CD16^+^ NK cells, respectively, throughout this review as it can be difficult to distinguish between CD56^br^ and CD56^dim^ subsets in lung tissue – particularly in lung tumors (28, 29). CD16^−^ NK cells are less differentiated and have low cytolytic ability, but produce greater amounts of IFNγ and TNFα than their CD16^+^ counterparts (30, 31). Conversely, CD16^+^ NK cells are more differentiated, with high cytolytic activity (30, 31) (**Figure 1**). A key difference between the two subsets in the blood is that CD16^−^ NK cells lack the expression of KIRs (31, 32). NK cell expression of KIRs is acquired during their maturation, in a process known as NK cell education or licensing, and expression of KIRs is vital for the cytolytic activity of NK cells (33–35). However, expression of KIRs by CD16^−^ NK cells does occur in some tissues, such as the lung (21, 36).

The lung microenvironment regulates resident immune cells at homeostasis to prevent unwanted activation by harmless antigens. Alveolar macrophages are a prime example of this; these cells are regulated by anti-inflammatory soluble factors such as TGFβ and IL-10, and by ligation of inhibitory receptors such as CD200R and SIRPα (37). The lung NK cell population as a whole (including circulating and tissue-resident subsets) in both humans and mice similarly displays hypo-functional features in the healthy state compared to NK cells from the blood or other organs (38–41). *In vitro*, human lung-derived NK cells exhibit 25-fold lower cytotoxicity against K562 cells (a cell line particularly sensitive to NK-cell-mediated lysis) (39) and decreased degranulation and ADCC responses compared to blood-derived NK cells (38). In mice, lung NK cells also show decreased cytotoxicity and degranulation versus NK cells isolated from the spleen (40), and express higher levels of the inhibitory receptor NKG2A and lower levels of activating receptors NKp44 and NKG2D (41). Therefore, lung NK cells are thought to be more tightly regulated with a higher threshold for activation than NK cells in other tissues.

In the blood (where they are known as circulating NK (cNK) cells) approximately 10% of NK cells are CD16^−^ and 90% CD16^+^ (31). The CD16^−^ compartment can also contain CD16^+^ NK cells that transiently lose expression of CD16 following activation, when CD16 is cleaved from the cell surface (42–45). In the lung, the distribution of CD16^+^ to CD16^−^ NK cells is similar to that in the blood (38, 46); however, it does vary, for example in the liver and gut, where the CD16^−^ population predominates (25, 26). In addition, recent studies show the presence of possible ‘resident’ NK cells in certain tissues (particularly the liver) on the basis of the expression of the markers CD69, CD103 (αE integrin) and CD49a (α1 integrin), which cause their retention (20, 47, 48) (**Figure 1**). CD69 interacts with sphingosine-1-phosphate receptor 1 (S1PR1) to promote its degradation; as a result, S1PR1 surface expression is decreased, causing a reduction in chemotactic cues to the blood where high levels of S1P are present. CD49a and CD103 have specific ligands: CD103 binds to E-cadherin, which is expressed by epithelial cells; and CD49a binds to collagen IV, a component of the ECM. For simplicity, we will refer to these cells as tissue-resident NK (trNK) cells in this review, but cells with this phenotype have also been described (in various tissues) as ILC1s (49–51), CD103^+^ ILC1s (50) and intra-epithelial ILC1s (ieILC1s) (2, 50, 52, 53). In the placenta there is also a population of NK cells that expresses CD49a and CD103 described as decidual NK cells (dNK cells) (2, 54). dNK cells have unique functions that are crucial for successful pregnancy which include maintaining maternal-fetal immune tolerance and promoting uterine spiral artery remodeling; the latter being necessary for allowing sufficient blood flow to both the placenta and the developing fetus (2, 54) [reviewed by Liu et al. (55)]. In both humans and mice, and between different organs, the distinction between trNK cells and ILC1s/ieILC1s is blurred, with few clear markers to distinguish these cell types and no clear differences in function [reviewed by O’Sullivan 2019 (56), and Peng and Tian 2017 (57)]. We have summarized the similarities and differences between trNK cells, ieILC1s and ILC1s in the lung specifically in **Table 1**.

**Table 1:** Characteristics of trNK cells, ieILC1s and ILC1s in the human and mouse lung.

	trNK	ieILC1	ILC1
**Human**			
**Markers** **(unstimulated)**	CD56+/bright CD16−/low CD69+ CD103+ CD49a+ Perforin −/low Granzyme B −/low CXCR6+ NKG2A+ CD57− CCL5+	CD56+ CD16- CD69+ CD103+ CD49a+ Perforin low Granzyme B low CD127− CD161+ NKp46+ CD94+ 2B4+ CD160+ CD122+	CD56+/− CD16− CD69+/− CD49a- IL-12RB2+ NKp44+/− CD117− CD127+ CD161+ CRTH2−
**Transcription factors**	Tbet+ Eomes+	Tbet+ Eomes+	Tbet+ Eomes+/−
**Response to stimulation**	**PMA/ionomycin:** Increased GM-CSF expression by CD49a^+^ trNK vs CD16^−^ cNK. ~60% of CD49a^+^ trNK express IFNγ and TNFα, but no difference in IFNγ or TNFα expression between CD49a^+^ trNK and CD16^−^ cNK. Low (10 - 30% positive) expression of CD107a, no difference in expression between CD49a^+^ trNK and CD16^−^ cNK. **IL-15:** Increased Ki-67 expression on CD16^−^ CD49a^+^ trNK (~40%) vs CD16^+^ cNK (~10%), but no difference compared to CD16^−^ cNK. 100% expression of perforin and 60% expression of granzyme B on CD49a^+^ trNK – less than CD16^+^ cNK (but no difference as compared to CD16^−^ cNK) **Ex vivo influenza X31:** Increased CD107a on CD16^−^ CD49a^+^ trNK vs CD16^−^ cNK (but no difference between CD16^+^ CD49a^+^ trNK and CD16^+^ cNK) No significant difference in expression of CD107a or TNFα between CD49a^+^ trNK and CD16^−^ cNK.	**IL-12 + IL-15:** ~10% positive for IFNγ (no difference between ieILC1 and CD103^−^ NK cells) **IL-12 + IL-18:** ~10% positive for IFNγ (no difference between ieILC1 and CD103^−^ NK cells)	**PMA/ionomycin:** Can produce IFNγ *(cannot say % positive as cells gated on total ILC population, not ILC1 specifically)*
**References**	(21, 26, 46, 58, 59)	(2)	(1, 60–64)
**Mouse**			
**Markers** **(unstimulated)**	Lineage − NK1.1+ CD49a+ CD49b− CD11b high CD27 low	Not described in the lung	Lineage − NK1.1+ CD49a+/high CD49b+/low CD11b+ CD27− CD90+ NKp46+ CD127−
**Transcription factors**	Not described in the lung	Not described in the lung	Tbet+ Eomes low/− RORgT−
**Response to stimulation**	Not described in the lung	Not described in the lung	**PMA/ionomycin:** ILC1 produced less IFNγ and TNFα than NK cells and were less cytotoxic
**References**	(48, 65, 66)	Not described in the lung	(4, 67, 68)

In the lung, trNK cells comprise 10–25% of the total NK cell population, with the overwhelming majority of trNK cells being CD16^−^ (46, 48, 58, 59). As the majority of the NK cells in the lung are circulating rather than tissue-resident, this provides an explanation for the predominance of CD16^+^ NK cells in this organ, whereas other tissues — namely the liver, skin and secondary lymphoid organs — have a larger population of CD16^−^ trNK cells (48). A caveat in the identification of trNK cells in the human lung is that all studies (21, 46, 58) use lung tissue obtained from patients with lung cancer (tissue that is distal from tumors). trNK cells (described as ieILC1-like cells) are also reported at a higher frequency in lung tumor regions than in adjacent non-cancerous lung tissue (2). A population of trNK cells is also present at low frequencies in the blood, although the frequency of these cells was increased in lung cancer patients compared to controls (21).

Whether lung trNK cells are truly resident remains unclear. In terms of their function, one study which separated CD16^−^ trNKs into CD56^br^ and CD56^dim^ trNK cells found that CD16^−^ CD56^br^ trNK cells have higher expression of the degranulation marker CD107a than CD16^−^ CD56^br^ cNK cells in response to influenza A virus in a human lung explant model (46). However, this difference is not observed between CD16^−^ CD56^dim^ trNK cells and CD16^−^ CD56^dim^ cNK cells. Additionally, no difference is observed in CD107a expression or production of granzyme B, IFNγ or TNFα between trNK cells and cNK cells in response to PMA and ionomycin (46, 58); however trNK do produce increased levels of GM-CSF and show decreased perforin expression (58). In addition, a sub-population of trNK cells described as adaptive-like trNK cells has recently been described in the human lung, characterized by expression of CD49a, NKG2C and KIRs (21). In line with their memory-like phenotype, these cells display greater cytokine production and cytotoxicity than CD49a^+^ NKG2C^-^ trNK cells (21). The discovery of these cells adds further confusion to the nomenclature surrounding trNK cells, and more research is needed to determine the similarities and differences between trNK cells and memory-like NK cells.

Differences are observed at the transcriptional level in lung trNK cells (in this study defined as CD69^+^CD49a^+^ and/or CD103^+^) compared to CD69^+^ single-positive and CD69-negative NK cells, with higher expression of *ID3*, *IRF4* and *RBPJ* in the former (58). Genes involved in tissue retention such as *RGS1*, *RGS2* and *ZNF683* (Hobit) are also upregulated in the trNK cells compared to CD69^−^ NK cells. Different tissue resident immune cell subsets share some features, for example, lung trNK cells express high levels of *CXCR6* and *RGS1*, and have reduced levels of expression of *SELL*, *S1PR5* and *FGFBP2* similar to lung and spleen CD8^+^ tissue-resident memory T cells (TRM) (58, 69). Liver trNK cells also express high levels of CXCR6 at both the mRNA and protein level (20) and produce high levels of IFNγ, TNFα and GM-CSF but lower levels of perforin upon stimulation; similar to trNK cells in the lung (70, 71).

Comparison of human NK cell subsets with those in mice are difficult as murine NK cells do not express CD56, but are instead identified using NK1.1 and/or CD49b (DX5), and with maturity determined using the expression of CD11b and CD27 (**Figure 1**) (72, 73). Fortunately, most activating and inhibitory receptors are conserved between human and mouse; for example, NKp46 and NKG2D (74). CD49a is also used as a marker for tissue residency in mice, however most studies refer to trNK cells as ILC1s as, in mice, the population tends to lack expression of the transcription factor Eomes (75). Despite these differences, parabiosis mouse models show that 80–90% of lung NK cells are cNK cells (4, 76), with the remaining 10–20% presumed to be trNK cells, in agreement with the human studies described above. Further studies are needed to ascertain the functional role of this potential trNK cell population.

## NK Cells in Disease Settings

From a historical perspective, as shown in their name as “killer” cells, the typical view has been that NK cells play a beneficial role in diseases: clearing infection and preventing development of tumors. However, it is now clear that there is much more nuance to this, and NK cells can have both beneficial and detrimental roles. Indeed, they can play an immunoregulatory role in restraining the activity of other immune cells at sites of infection (77), although in the tumor setting this is unclear. Their recruitment and function in disease depends on the tissue microenvironment (e.g. extracellular matrix), the level of tissue damage and the general inflammatory milieu – which rises and falls quickly in lung infection, but is slower to rise and becomes chronic during tumor development (78).

### NK Cells in Pulmonary Infections

In the healthy lung, NK cells are localized to the interstitium rather than to the airways (38). However, NK cells are observed in the bronchoalveolar lavage (BAL) fluid after infection with influenza virus (79), *Staphylococcus aureus* (80), cytomegalovirus (CMV) (81), and SARS-CoV-2 (82). NK cells are also present in BAL fluid in various non-infectious diseases, such as asthma and chronic obstructive pulmonary disease (COPD) (47).

Despite an apparent hypo-functionality at homeostasis, NK cells respond and activate quickly in response to pulmonary infections. In humans, rare genetic disorders affecting NK cell function are associated with increased respiratory infections, particularly viral infections (13, 83). As ‘poised effector’ cells, NK cells are early responders to infection and animal models show that substantial numbers are recruited to the lungs 2–3 days after infection with influenza virus, *S. aureus* and *Klebsiella pneumoniae* (41, 84, 85). The increase is thought to be due to NK cell recruitment rather than local proliferation, as the numbers of splenic and circulating NK cells show concomitant decreases following infection (41, 86). This increase is transient, as levels return to normal by day 6–9 after infection (85, 87). Similarly in SARS-CoV-2 infection in humans, peripheral NK cells are reduced during acute infection (88–90) and return to normal levels as the infection is cleared; however, in patients with severe COVID-19 the NK cell count remains low at 3 weeks post infection (91).

**Figure 2:**
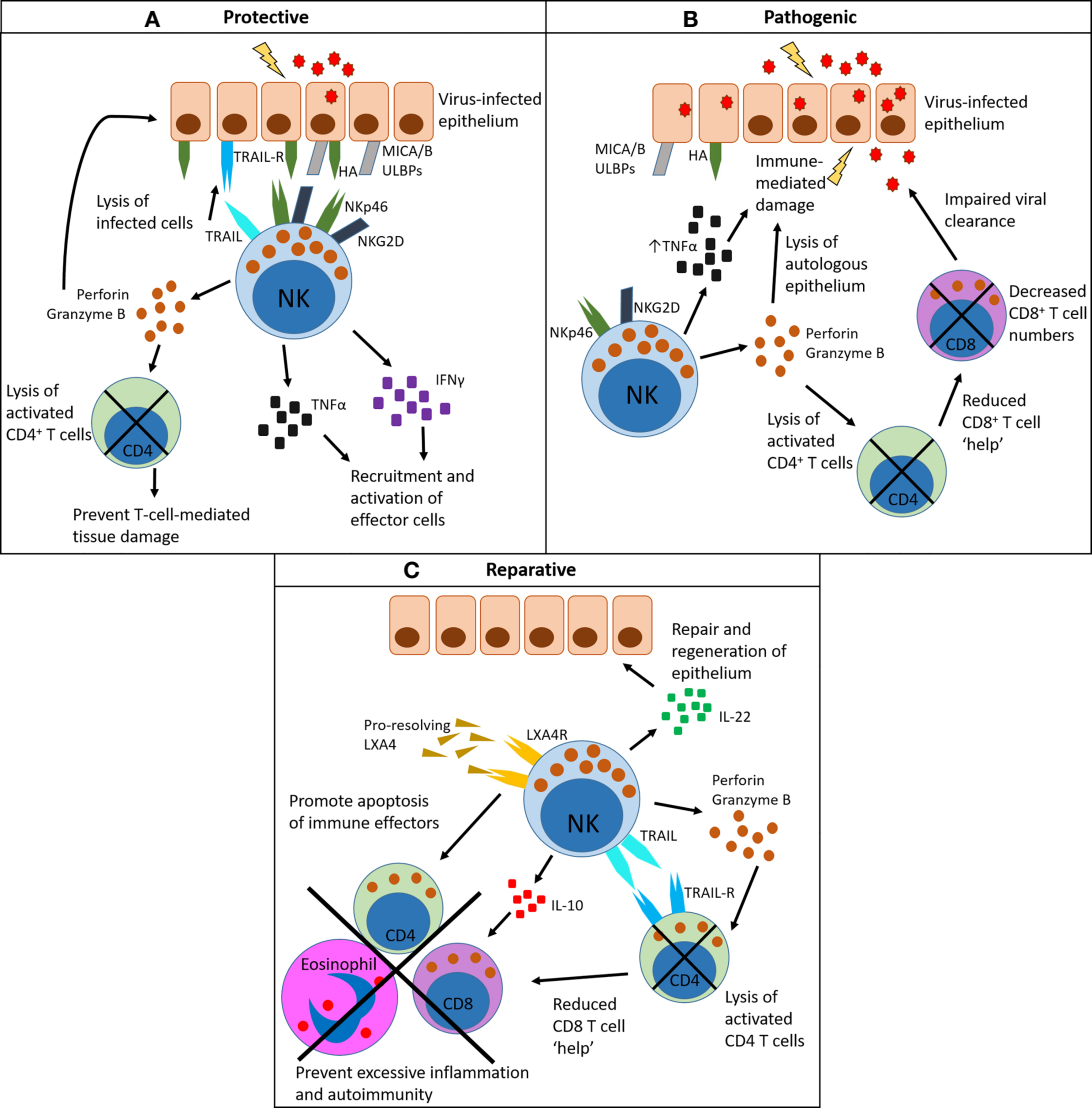
Pleiotropic functions of NK cells in the lung: protective, pathogenic and reparative. **(A)** NK cells have a protective role by recognizing virally infected cells that display viral proteins on their surface (for example, HA) and/or upregulate damage-associated molecules (for example, MICA/B and ULBPs). These bind to activating receptors on NK cells such as NKp46 (which binds HA) and NKG2D (which binds MICA/B and ULBPs). Once activated in this way, NK cells produce the inflammatory cytokines TNFα and IFNγ, which aid recruitment of effector cells to the site of infection and facilitate their activation. They also produce perforin and granzyme B, which directly lyse infected cells, and can mediate the contraction of activated CD4^+^ T cells to prevent immune-mediated tissue damage. Lysis of infected cells can also be death-receptor-mediated, *via* TRAIL or FasL. **(B)** NK cells can drive a pathogenic outcome in infection, particularly in cases of high viral load. Here activated NK cells produce excessive amounts of TNFα, which damages the epithelium and causes excessive recruitment of other immune cells. Excess lysis of activated CD4^+^ T cells through the release of perforin and granzyme removes the help for other immune cell subsets, particularly virus-specific CD8^+^ T cells, which results in impaired viral clearance. **(C)** NK cells can mediate the resolution of inflammation following infection. They are a source of IL-22, which promotes repair of the epithelium. They express the receptor LXA4R, which binds to the pro-resolving mediator LXA4. As a result of LXA4 binding, NK cells promote the apoptosis of immune effector cells such as CD8^+^ T cells and eosinophils. They also clear activated CD4^+^ T cells in a TRAIL-dependent manner, reducing CD8^+^ T cell help. NK cells also produce IL-10 which has general anti-inflammatory activity. Together, these clearance mechanisms prevent prolonged inflammation after infection.

Activation of NK cells in the lung is facilitated by the upregulation of DAMPs, which are often ligands of NK cell activating receptors, particularly NKG2D (92). For example, *Mycobacterium tuberculosis*-infected monocytes and macrophages upregulate the NKG2D ligand ULBP1, and blocking ULBP1 prevents NK cell-mediated lysis of infected mononuclear cells (93). Airway epithelial cells also express the NKG2D ligands MICA and MICB (referred to as MICA/B hereafter) and ULBPs under stress (94). In addition, NK cells bind directly to viral proteins expressed on the surface of infected cells, such as the haemagglutinin (HA) glycoprotein of influenza virus, which is recognized by the activating receptor NKp46 (95). Once activated, NK cells produce pro-inflammatory cytokines including TNFα and IFNγ. The latter has a number of important roles, including induction of interferon-stimulated genes in nearby cells (96), recruitment of other immune cells to the site of infection, and aiding the activity of CD8^+^ T cells (34, 83). In addition to cytokine production, activated NK cells also release perforin and granzymes, which induce apoptosis in targeted cells (**Figure 2A**). NK cells can also kill virally infected cells through death-receptor-mediated pathways; for example, influenza A induces the expression of TRAIL on NK cells, and blocking TRAIL *in vivo* results in reduced viral clearance (97).

Animal models show that lung NK cells have both protective and damaging roles in infection. In influenza virus and *S. aureus* infection, mice that lack NK cells (*Ncr* ^−/−^, *Il15* ^−/−^, or depleted using anti-NK1.1 antibodies) show a higher lung viral or bacterial burden at the peak of infection and a longer infection period (46, 80, 86). However, NK cells can also cause harm by contributing to inflammatory-mediated damage, particularly with high dose infections (85–87, 98) [reviewed by Frank and Paust 2020 (99)]. In a more regulatory role, in some tissues NK cells can also indirectly inhibit viral clearance by promoting the apoptosis of activated CD4^+^ T cells, which in turn reduces CD8^+^ T cell numbers (**Figure 2B**). However, in the same way, decreasing CD8^+^ T cell numbers can also prevent CD8^+^ T cell-mediated immunopathology (**Figure 2C**) (100). Therefore, timely induction of NK cell activation — but also contraction of their responses — is optimal for virus containment. Impaired NK cell responses occur in aged mice, which show decreased numbers in the lung following infection and reduced levels of the activation marker CD69 (101). Clearly, NK cells are important in the lung, but their impact on the outcome of an infection in mice is affected by factors such as viral titre and the strain and age of the mouse (85, 98, 101).

#### NK Cells in the Resolution of Inflammation

As with most immune cells, NK cells have pleiotropic functions and are involved in the resolution of inflammation. As described above, NK cells can lyse activated CD4^+^ T cells *via* perforin and granzyme, thereby removing 'help' for CD8^+^ T cells (100). NK cells can also directly lyse activated CD8^+^ T cells, which again can have both pathogenic (impaired viral clearance) and pro-resolving (prevent immunopathology) effects; although this has not been shown specifically in the lung (102, 103). However, in an influenza infection model, antigen-specific CD8^+^ TRM are significantly raised in NK cell-depleted mice, providing an improved response to re-infection with a different influenza strain; implying that NK cell-mediated clearance of CD8^+^ T cells in the lung may have detrimental effects in the long term (104).

Resolution of inflammation is also facilitated by NK cell production of IL-10 that limits anti-viral CD8^+^ T cell responses (105), NK cell expression of TRAIL that may facilitate removal of neutrophils and activated CD4 T cells (106, 107), and, in the case of asthma, lysis of granulocytes and T cells in an NKG2D-dependent way (108–110). Indeed, impaired NK cell-mediated killing is associated with severe asthma (110). NK cells also express receptors for the pro-resolving mediator lipoxin A4 (LXA4) (109, 110), which upon binding promotes NK cell-driven apoptosis of eosinophils and neutrophils through NKG2D (108). Furthermore, depletion of NK cells delays the resolution of allergic airways disease in mice (108). NK cells have also been described as a source of IL-22 in the lung following influenza virus infection in mice, which is crucial for the repair and regeneration of the tracheal epithelial layer after severe infection (79) (**Figure 2C**). However, more recent studies show non-NK ILCs (ILC3s) as a more important source of IL-22 in the lung (111, 112). Zwirner, Domaica and Fuertes have recently reviewed the regulatory functions of NK cells in detail (77). As such, it seems that NK cells have diverse roles in the lungs, beyond their activity as killer cells.

NK cells are therefore protective, pathogenic and reparative in lung infection (85, 86, 98, 113, 114) (**Figure 2**). The molecular mechanisms that govern each role are at present unclear, but they are clearly driven by the local tissue microenvironment. A caveat here is that the vast majority of research discussed above is derived from mouse models. Further study is needed to determine if NK cells in the human lung have the same roles.

### NK Cells in Fibrotic Lung Disease

NK cell activity will alter depending on the physical and chemical properties of the tissue. These changes in tissue architecture are particularly evident in fibrotic lung diseases associated with chronic inflammation, and alterations in NK cell function are seen in a number of fibrotic lung diseases. For example in idiopathic pulmonary fibrosis (IPF), decreased expression of the activating receptor NKG2D on NK cells (and NKT cells and γδ T cells) isolated from the BAL fluid has been documented, indicating a decrease in NK cell functionality in IPF (115). In addition, NK cells are reduced in the blood of IPF patients (116). In COPD, which also contains a fibrotic element, lung NK cells have increased cytotoxic activity in comparison to NK cells from similar patients without COPD, and may actually drive destruction of the epithelium in COPD (113, 117). In a mouse model of pulmonary fibrosis (bleomycin-induced), the disease is more severe in mice where CXCR3-dependent immune cell (particularly NK cell) recruitment is reduced, resulting in increased mortality in *Cxcr3^-/-^* animals (118). Indeed, IFNγ was shown to have a protective effect in this model (118), implying that NK cells as a key source of IFNγ could have a beneficial role in pulmonary fibrosis, in contrast to the studies described above in COPD. This is an understudied area - NK cells can have negative and positive consequences in different fibrotic lung diseases.

### NK Cells in Lung Cancer

Abundant NK cells or a dominant NK cell gene signature within a tumor correlates with better overall survival in a number of cancers, including lung cancer [reviewed by Larsen, Gao and Basse (119)], but the prognostic importance of NK cells in lung cancer is unclear. Some studies link high tumor NK cell infiltration with increased survival (120, 121), whereas others show no correlation (29, 122). Early studies grouped NK cells, NKT cells, γδ T cells and/or ILC1s together [reviewed by Habif et al. (122)] which has now been largely overcome by using antibodies to NKp30 (36) or NKp46 (25, 29), or by co-staining of CD45^+^ CD3^−^ CD56^+^ cells (122). Discrepancies also arise owing to the different stages of tumor development examined and the precise microenvironment generated (78). Regardless of their prognostic significance, it is clear by both immunohistochemistry and flow cytometry that lung tumors have low NK cell infiltration (25, 28, 29, 36, 122–124). Additionally, NK cells tend to locate at the tumor edge, or in the tumor stroma, indicating a possible defect in their recruitment (25, 29, 36, 122). Factors affecting NK cell recruitment to both tumors and sites of infection include chemokines (125, 126), extracellular matrix (127, 128) and soluble mediators (129, 130), and these will be discussed in the next section.

**Figure 3:**
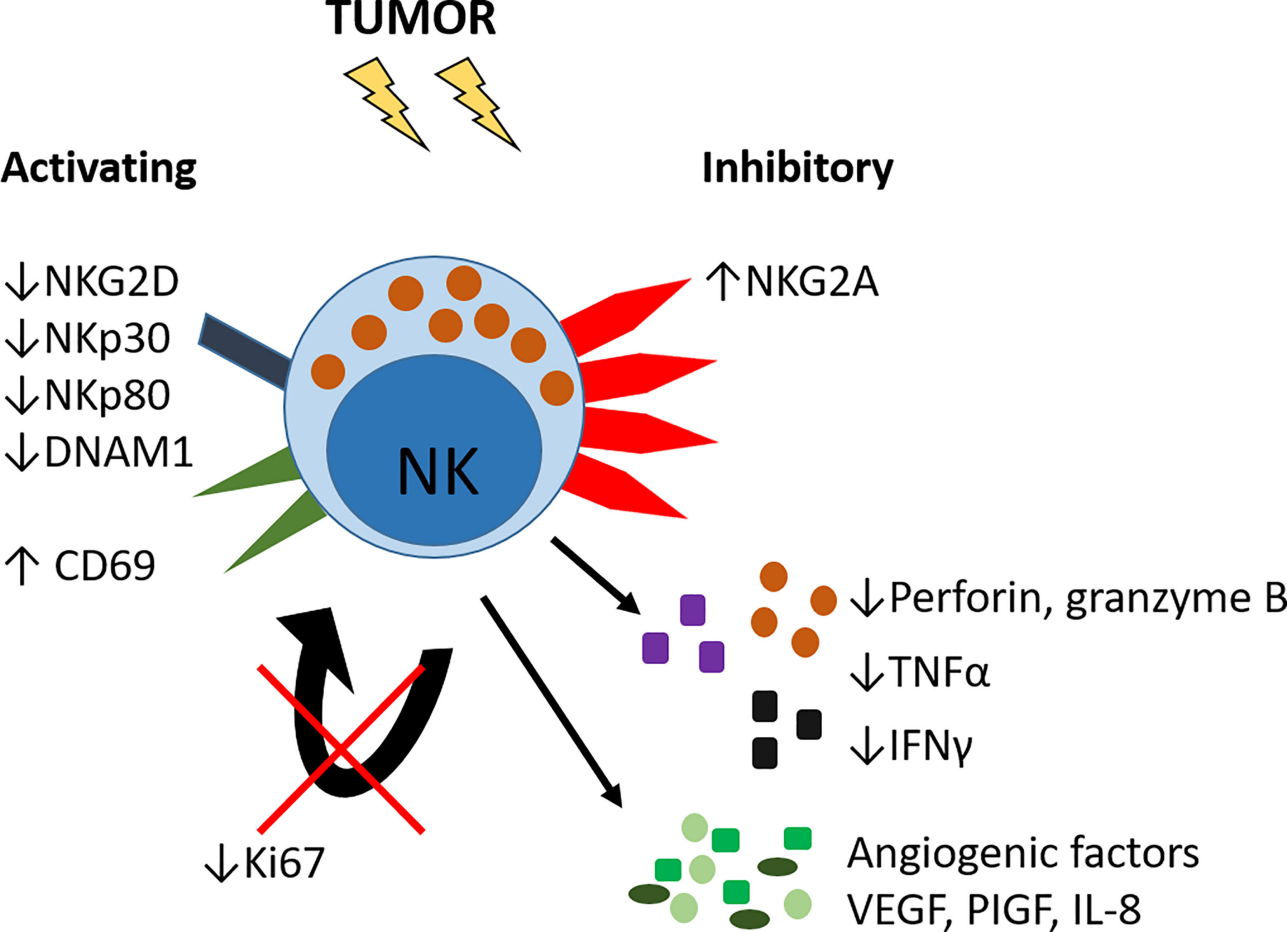
Altered phenotype of lung tumor NK cells. NK cells in lung tumors have reduced expression of activating receptors including NKG2D, NKp30, NKp80 and DNAM1 and increased expression of the inhibitory receptor NKG2A. The activation marker CD69 is increased. Tumor NK cells have reduced expression of Ki67 and hence reduced proliferative capacity, and decreased expression of pro-inflammatory cytokines TNFα and IFNγ, and decreased production of perforin and granzyme B. Lung tumor NK cells can also produce pro-angiogenic growth factors VEGF, PlGF and IL-8.

As with viral infection, animal models of lung cancer indicate that the timing of NK cell infiltration may be crucial for their anti-tumor activity. In a mouse model of lung cancer, depletion of NK cells at early stages of cancer — but not at later stages — accelerated tumor growth (131). Furthermore, as tumor growth progresses, both mouse and human NK cells exhibit decreased expression of the degranulation marker CD107a (29, 36, 131), as well as decreased IFNγ (29, 131) and perforin (36, 131) (**Figure 3**). In mouse studies only, tumor NK cells have decreased expression of granzyme B and reduced proliferation (131) (**Figure 3**). In lung adenocarcinomas, as well as in other solid tumors, intratumoral NK cells are predominantly CD16^−^, rather than the more cytotoxic CD16^+^ subset seen in the neighbouring healthy margin tissue (29, 36, 122–124). Furthermore, downregulation of activating receptors, including NKG2D [as is also seen in IPF (115)], and upregulation of the inhibitory receptor NKG2A, reduce anti-tumor potency (28, 29, 122) (**Figure 3**). As mentioned previously, CD16 is enzymatically cleaved from NK cells after receptor engagement and activation (42, 43) and re-expression can be delayed (44, 45) meaning that NK cells in lung tumors may be ‘ex-CD16^+^’ rather than bona fide CD16^−^ NK cells. NK cells isolated from lung tumors (as well as matched blood and lung margin) have also been shown to produce tumor-promoting angiogenic factors, including vascular endothelial growth factor (VEGF), placental growth factor (PlGF) and IL-8 (**Figure 3**) (123). Although there was no significant difference in the expression of these factors between tumor, lung margin and blood NK cells, it is interesting to note that CD16^−^ NK cells – which are present in greater abundance in lung tumors – produce far greater amounts than CD16^+^ NK cells (123).

This population of CD16^−^ NK cells in lung tumors may also comprise trNK cells, which, in the lung, are predominantly CD16^−^ (58). To support this idea, tumor NK cells express higher levels of CD69 compared to unaffected lung or blood NK cells (29, 36, 132); a marker highly expressed on trNK cells (38, 46, 58). Indeed, a population resembling trNKs (classified as ieILC1s) is apparent in human lung tumor tissue (2). Further characterization of tissue residency markers is required to determine what contribution trNK cells make to the intratumoral NK cell compartment.

## Factors Influencing Lung NK Cell Recruitment and Function

The process of NK cell recruitment into the infected or malignant lung is very similar, though the magnitude will depend on the extent and nature of tissue damage. Affected areas have altered cellular composition, including changes in the phenotype, function and numbers of fibroblasts, stem cells, endothelial cells and immune cells including macrophages and lymphocytes. As such, many soluble factors are present at high levels in the infected lung and in the lung TME that are not normally expressed or expressed at low levels within healthy tissues: for example, chemokines, hypoxia-inducible factors and TGFβ (129, 130).

### Chemokines

cNK cells express a broad repertoire of chemokine receptors depending on their differentiation stage (25, 133) (**Table 2**), providing one explanation for the variation in the ratio of CD16^−^ to CD16^+^ NK cells in different tissues. For example, CD16^−^ NK cells express CCR7 (and CD62L) which promote recruitment to secondary lymphoid tissues (25, 133, 134). Furthermore, organ-specific chemokine patterns exist in the steady state and in disease (135).

**Table 2:** Chemokine receptor expression on human NK cell subsets, and their corresponding chemokine ligands.

CD16^−^ CD56^br^		Both		CD16^+^ CD56^dim^	
Receptor	Ligand(s)	Receptor	Ligand(s)	Receptor	Ligand(s)
CCR7	CCL19, CCL20	CXCR4	CXCL12	CX3CR1	CX3CL1
CCR5	CCL3–5	CXCR3	CXCL4, CXCL9-11	CXCR2	CXCL1-3, CXCL5
				CXCR1	CXCL8

Although CXCR3 can be expressed by both CD16^−^ and CD16^+^ NK cells, it is predominantly expressed by CD16^−^ NK cells [~10% of CD16^+^ NK cells express CXCR3 vs ~90% of CD16^−^ NK cells (136)] and therefore is more important for mediating their recruitment (25, 136). At steady state, *Cxcr3* ^−/−^ mice have decreased NK cells in the lungs, liver, blood and lymph nodes (118, 137) and fewer NK cells are recruited during lung infection (125, 138). In the peripheral blood of influenza and COVID-19 patients, fewer CXCR3^+^ CD16- NK cells are present (136), presumably because these cells have been recruited to the lung. In accordance with this, the expression of *CXCL10* (a ligand for CXCR3) is increased in the BAL fluid of SARS-CoV-2 infected patients, and NK cells isolated from the BAL fluid of COVID-19 patients also express high levels of *CXCR3* (82, 136). CCR5 is also implicated in NK cell recruitment to the lung, although to a lesser extent than CXCR3. *Ccr5* ^−/−^ mice only show a small reduction in pulmonary NK cell numbers after influenza infection (125). *CCL5* expression is also increased in the BAL fluid of COVID-19 patients (136).

CXCR3 and its ligands CXCL9-10 also mediate the recruitment of NK cells into tumors. In subcutaneous and pulmonary mouse tumor models (RMA-S, a lymphoma tumor cell line expressing low levels of class I MHC; B16 melanoma cell line transduced to express the NKG2D ligand RAE-1ϵ; and pulmonary tumor cell line 3LL-Luc2), decreased intra-tumoral NK cells are observed in *Cxcr3^-/-^* mice and in mice treated with anti-CXCR3 antibodies; but despite this, no difference in survival is observed (65, 126). However, subcutaneous injection of tumor cells transduced to overexpress CXCL10, which results in increased NK cell tumor infiltration, does increase overall survival as compared to control cell injection (106). Tumor metastasis to the lung is facilitated by total NK cell depletion, but not by anti-CXCR3 antibodies, implying a role for other chemokine receptors (65). For example, IL-33 can drive the production of CCL5 from eosinophils and CD8^+^ T cells, which significantly increases survival and decreases lung metastases in an NK cell-dependent manner (139).

Chemokine production in infected or malignant tissue is also affected by other soluble factors. For example, IFNγ increases the production of the CXCR3 ligands CXCL9-11 by tumor cells, and correlates with an increase in NK cells in subcutaneous tumors and improved survival in mice (126). However, the immune modulator and tumor-promoting factor prostaglandin E2 (PGE2) inhibits IFNγ-induced secretion of CXCR3 ligands from breast cancer cell lines (140). Similarly, IFNγ-matured dendritic cells (DCs) exhibit reduced production of CXCL10, CCL5 and CCL19 in the presence of PGE2, which results in reduced NK cell migration *in vitro* (141).

**Figure 4:**
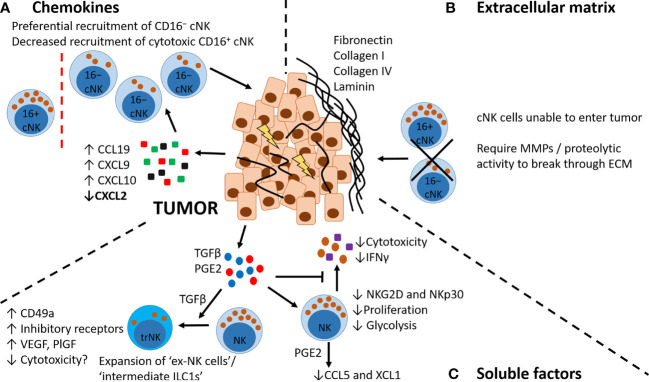
Factors affecting NK cell recruitment to tumors. **(A)** Chemokines. Tumor cells and immune cells in the TME produce increased levels of CCL19, CXCL9 and CXCL10 and decreased levels of CXCL2, which may preferentially recruit CD16−NK cells expressing CCR7 and CXCR3. **(B)** Extracellular matrix. Tumors can be surrounded by a dense ECM layer that restricts or prevents NK cell entry from the circulation. Entry requires specific proteolytic activity, e.g. MMPs. **(C)** Soluble factors. Tumor cells and immune cells in the TME secrete soluble factors including TGFβ and PGE2. TGFβ can promote a phenotypic switch in NK cells to a more trNK cell-like phenotype (also known as ex-NK cells, and intermediate ILC1s), with increased expression of CD49a, increased production of pro-angiogenic factors VEGF and PlGF, and increased expression of inhibitory receptors. These trNK cells may also have decreased cytotoxicity. TGFβ and PGE2 have other inhibitory effects on NK cells, including reducing NK cell cytotoxicity, decreasing inflammatory cytokine production, decreasing proliferative capacity and altering their metabolic activity (decreasing glycolysis). PGE2 can also alter NK cell chemokine production, decreasing secretion of CCL5 and XCL1.

In some instances, it is not NK cell recruitment that is the problem, but the recruitment of inappropriate NK cell subsets. For example, increased levels of CCL19, CXCL9 and CXCL10 in lung tumors may preferentially recruit CD16−NK cells, and reduced levels of CXCL2 may decrease recruitment of more cytotoxic CD16^+^ NK cells (**Figure 4A**) (25). These CD16^−^ NK cells may also be inappropriately retained *via*, for example, reduced expression of S1PR1 that is needed for tissue egress, and increased expression of CXCR6, promoting retention (132).

Clearly, therapeutic targeting of specific chemokine axes may facilitate the recruitment of cytotoxic CD16^+^ NK cells to tumors, but the potential for off-target effects remains a caveat. Genetic modulation of chemokine receptors is a current field of research for NK cell-based therapies, and provides a more targeted method of altering the chemokine axes of NK cells (142, 143). However, altered chemokine axes cannot explain all defects in the recruitment of NK cells, as in some cancers CX3CL1 (the ligand for CX3CR1) expression is high but CD16^+^ NK cells are not present, despite expressing CX3CR1 (144).

### The Role of Matrix

Alteration of the extracellular matrix (ECM) occurs in viral infections (145), and in cancer (78) and may affect NK cell recruitment, retention and activation.

In both animal tumor models and human cancers, tumors can be surrounded by ECM, with some encapsulated in laminin or collagen (**Figure 4B**) (146–148). Indeed, lung tumors are surrounded by type I collagen (149), and small cell lung cancers (SCLC) and their metastases are enveloped in a dense ECM composed of fibronectin, laminin, collagen IV and tenascin C (150). Additionally, tumor resistance to PD-1/PD-L1 blockade is associated with increased collagen deposition (151). It has also been observed in influenza mouse models that ECM alterations, which are observed in acute infection, can persist well after viral clearance (145). To migrate through tissues, particularly those with dense ECM, many cells (including NK cells) release proteases such as MMPs (152). In the Lewis lung carcinoma (LLC) mouse model, tumors with more altered structural properties have less NK cell infiltration compared to tumors with a high NKp46, IFNγ and, somewhat controversially, fibronectin signature (127). Furthermore, *Ifnγ* ^-/-^ mice have primary tumors with a more aggressive, ECM-enriched phenotype, and increased metastases (127). Fibronectin and other ECM proteins are generally associated with more mesenchymal, invasive tumors. However, the presence of fibronectin in tumors can be beneficial if accompanied by a decrease in other typical epithelial-to-mesenchymal transition (EMT) markers including keratin, vimentin and N-cadherin (127). It should also be noted, that cross-linking and density of tumor ECM influences NK cell recruitment with ‘loose’ facilitating and ‘compact’ inhibiting (128). The role of ECM in NK cell recruitment to lung tumors, and indeed in recruitment to the fibrotic lung, is an emerging area of research.

### TGFβ and PGE2

The soluble mediators TGFβ and PGE2 inhibit immunity in the healthy lung but also impair immunity in the post-viral lung and in lung cancer (37, 78).

TGFβ has a number of immunoregulatory effects on NK cells *in vitro* including a decrease of activating receptors NKG2D and NKp30; a reduction in IL-15- or IL-2-induced NK cell cytotoxicity, proliferation, degranulation and granzyme B expression; a reduction of CD16-induced production of IFNγ; a decrease in glycolysis; and enhanced expression of the pro-angiogenic factors VEGF and PlGF (**Figure 4**) (10, 11, 123, 131, 153–155). With prolonged treatment, TGFβ also reduces NK cell-dependent ADCC responses (156). In mouse models of lung metastasis (B16 melanoma model, which spontaneously metastasizes to the lung), TGFβ promotes the expansion of an ‘intermediate ILC1’/’ex-NK cell’ population, associated with increased expression of inhibitory receptors, reduced cytotoxicity, and expression of the trNK-associated marker CD49a (**Figure 4C**). Interestingly, this population increases with tumor burden, as do the levels of TGFβ in the TME (157–159). *In vitro* studies with human blood-derived NK cells have also shown that TGFβ alone or in combination with IL-15 can promote the development of a trNK cell-like phenotype (53, 129, 153). However, unlike the studies described above in mice, these cells were highly cytotoxic (53). TGFβ (alone, or in combination with hypoxia and a demethylating agent) has also been shown to promote cNK cell acquisition of a dNK cell-like phenotype *in vitro* (160, 161). However in these studies, only the combination treatment resulted in a decrease in NK cell cytotoxicity (161). In the TME more broadly, TGFβ affects the chemokine milieu (78). For example, in a murine model of lung metastasis TGFβ suppressed the production of CXCL1 and CXCL5 by tumor cells (162) – chemokines associated with recruitment of CD16^+^ NK cells (36, 133).

PGE2 is a potent immune modulator produced during inflammation that, as mentioned previously, indirectly affects NK cells by reducing the levels of chemokines necessary for their recruitment (140, 141). PGE2 also directly suppresses NK cell proliferation, cytokine secretion, and cytotoxicity (**Figure 4C**) (141, 163, 164). In mouse tumor models (B16 melanoma and MC38 colorectal), a lack of the prostaglandin receptors EP2 and EP4 specifically on granzyme B^+^ cells – predominantly NK cells – causes tumor regression (165). PGE2 reduces the expression of the NK cell-derived chemokines CCL5 and XCL1 (**Figure 4C**), thereby decreasing the recruitment of conventional type 1 DCs to tumors (130, 166). In human immune cell datasets, *XCL1* and *XCL2* (homologues in humans for XCL1 in mice) are most highly expressed by CD16^+^ NK cells — the cells that are missing from the lung TME (130).

### Soluble NKG2D Ligands

The ability of NK cells to become activated depends on the expression of cell-surface receptors, for example, NKG2D (167, 168). However, activating receptors can be internalized or cleaved leading to cancer and viral escape (169–171). For example, MMPs and A disintegrin and metalloproteinases (ADAMs), which are often present in the TME (172) and the post-viral lung (173), cleave NKG2D ligands (NKG2DLs) from the cell surface of tumor cells or infected cells, which leads to the evasion of NK cell-mediated killing (114, 174–176). Increased levels of soluble NKG2DLs are observed in the TME and serum of patients with cancer, including lung cancer (177), which correlate with decreased overall survival (177–181). Similarly in IPF, increased levels of soluble MICA are observed in the plasma, and polymorphisms in MICA are associated with increased risk for the development of IPF (115). Soluble NKG2DLs inhibit both CD8^+^ T cells and NK cells as they block ligation of membrane-bound NKG2DLs (180, 182, 183) and cause downregulation of NKG2D (182, 183). NK cells with reduced membrane expression of NKG2D exhibit impaired lytic ability (183, 184), and mice that lack NKG2D are more susceptible to tumors (185). In various mouse models, prevention of tumor cell shedding of NKG2DLs increases the anti-tumor activity of NK cells and decreases the number of tumor metastases (175, 186). Additionally, combining anti-soluble MIC treatment with PD-L1 blockade improves survival in mice (186), and therefore highlights a potential future therapeutic opportunity for human cancers – particularly those with high serum levels of soluble NKG2DLs.

### Hypoxia

Another feature that influences NK cell activity is hypoxia, which occurs when the growth of tumor cells outstrips their supply of oxygen. In addition, the dense nature of the ECM around and within tumors can act as a barrier to critical metabolites (148). Hypoxia is often accompanied by lower pH and glucose levels (172). *In vitro*, hypoxia alone (1% O_2_) does not significantly affect NK cell cytotoxicity in comparison to normoxia (around 6% O_2_) (187–189). However, anoxia (0%) decreases NK cell-specific lysis of target cell lines *in vitro* (189). Hypoxic NK cells lose the ability to upregulate various activating receptors and produce less inflammatory cytokines in response to stimuli (190, 191); however, they can produce VEGF to promote angiogenesis (129). Additionally, indirect NK cell inhibition also occurs in hypoxic conditions, as hypoxia is associated with an increase in the shedding of NKG2DLs from cancer cells (192). Decreased pH levels also suppress NK cell cytotoxic activity and cytokine production (189, 193). A profound decrease in NK cell cytotoxicity occurs with a combination of low O_2_, pH and glucose *in vitro*, mirroring the conditions of the TME (189).

### NK Cell Interactions With Other Immune Cells

In tissues – whether they be trNK cells or recruited cNK cells – NK cells interact with many different immune cells (194), epithelial and endothelial cells (94, 117, 171). Lung trNK cells express different integrins to cNK cells (46, 58), therefore different NK cell subsets likely have different positions within the lung tissue and hence different cell types that they interact with.

**Figure 5:**
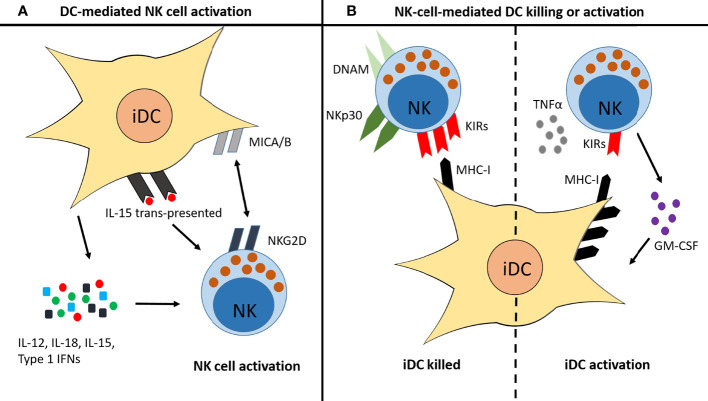
Reciprocal interactions between NK cells and immature DCs. **(A)** iDCs can promote NK cell activation and maturation in several ways: they secrete IL-12, IL-18, IL-15 and type I IFNs; they trans-present IL-15 to NK cells; and they express MICA/B, which binds to NKG2D on the surface of NK cells. **(B)** Once activated, NK cells then promote either killing of the iDC or activation of the iDC, depending on various factors. iDC killing is usually induced when the NK cell expresses high levels of NKp30 and DNAM, and/or when the iDC expresses low levels of MHC-I. iDC activation is induced when high levels of TNFα are present, when the NK cell produces GM-CSF, and/or when the iDC expresses high levels of MHC-I.

NK cell interactions with DCs are well studied (39, 40, 46, 117). Some interactions enhance NK cell activity, such as the interaction of CD16^−^ NK cells with immature DCs (iDCs) during infection. These iDCs secrete a number of cytokines, including IL-12, IL-18, IL-15 and type I IFNs, which promote NK cell maturation, activation and memory-like responses (**Figure 5A**) (117, 194–197). There are also contact-dependent mechanisms of DC-mediated NK cell maturation (**Figure 5A**) (195, 197). Such interactions are often reciprocal: once primed by iDC interaction, the activated NK cell then either induces killing of the iDC or its maturation (196, 197). Factors affecting this outcome are shown in **Figure 5B** (34, 194, 198). Environmental factors change the interaction between NK cells and DCs, for example cigarette smoke exposure in mice enhances NK cell cytotoxicity in a DC-dependent manner (117). A similar priming effect of DCs on NK cells is observed in COPD (117) that culminates in enhanced NK cell-mediated destruction of lung epithelial cells (113, 117). However, much less is known about NK cell-DC interactions in the TME.

In addition to DCs, NK cells are known to interact with macrophages. In pulmonary infection, macrophages increase their surface expression of NKG2DLs, in some cases rendering them susceptible to NK cell-mediated lysis (94, 199) [reviewed by (Stojanovic, Correia and Cerwenka, 2018 92)]. Tumor macrophages can also display increased levels of NKG2DLs (92, 200, 201), which are postulated to act on NK cells in a similar way to soluble NKG2DLs – in that low-level engagement of NKG2D promotes receptor internalization and desensitization of NK cells (92, 170). As with soluble NKG2DLs in serum, elevated expression of NKG2DLs is also observed on circulating monocytes in some cancers (201). Interestingly, this expression decreases following tumor resection surgery (201). *In vitro* studies show that the hypoxia-related factor lactate dehydrogenase 5 (LDH5) is able to drive expression of NKG2DLs on monocytes (201); hypoxia may therefore affect NK cell–macrophage communication. Additionally, tumor- or metastasis-associated-macrophages can express membrane-bound TGFβ (66), which will likely have similar inhibitory effects on NK cells as those described earlier for soluble TGFβ.

## Outlook: NK Cell-Based Therapies for Lung Cancer

Response rates to traditional chemotherapy and radiotherapy are poor in lung cancer, with lung cancer patients having a low 5-year survival rate of between 10-20% (202). Chemotherapies, radiotherapies and immunotherapies affect NK cell function; summarized in **Box 1**. T-cell-based immunotherapies target inhibitory molecules that are also expressed by NK cells, but their effect on NK cells is much less studied. In non-small cell lung cancer (NSCLC) (the most common lung cancer subtype), PD-1 and PD-L1 inhibitors are increasingly being used, and do improve survival in comparison to traditional chemotherapies (214, 215). However, the response rate is still only ~20%, with the greatest survival benefit seen in patients with tumors expressing highest levels of PD-L1 (214, 216). As such, a better understanding of how these therapies change NK cell activity may help to identify more effective dosing regimens and treatment combinations that promote NK cell tumor killing. In addition, specifically targeting NK cells with immunotherapies and/or cytokine therapies is also an area of expanding research (215). For example, targeting NK cell activation with an IL-15 therapy has shown early promise in clinical trials for NSCLC, in combination with PD-1 inhibition (217).

**BOX 1 | Current and future cancer therapies and their effects on NK cells**

Clinically, chemotherapies mainly suppress NK cell-mediated killing and cytokine production, although the effect varies depending on dose (203, 204). The mechanisms behind this suppression are poorly understood, as *in vitro* a number of chemotherapies actually enhance the immunogenicity of cancer cells to NK cells [reviewed by Zingoni et al. 2017 (203)]. NK cell recruitment may also be affected by chemotherapeutic drugs, as they can increase the expression of CXCR3 ligands in both human and mouse cancers (205).

Radiotherapy or ionizing radiation (IR) increases the expression of NKG2DLs and death receptors on cancer cells, making tumor cells more susceptible to NK cell-mediated lysis [reviewed by Chen et al. (206)]. However, IR also increases the cleavage of NKG2DLs from tumor cells (206), enabling the evasion of NK cell-mediated killing (174, 175). The effect of IR, like chemotherapy, is also dose-dependent, with low doses generally being beneficial for NK cell activity whereas high doses are detrimental (206).

Immune checkpoint receptors on T cells targeted by immunotherapies (for example, PD-1, TIGIT and CTLA-4) are also expressed by NK cells, therefore immunotherapies will also affect their activity. NK cells also contribute to the efficacy of monoclonal antibody therapies through the engagement of CD16, enabling the killing of target cells by ADCC (207, 208). New immunotherapies are in development to target specific NK cell inhibitory receptors, including anti-KIR and anti-NKG2A [reviewed by Sun H. and Sun C. (209)]. Indeed, targeting both NK cells and T cells can be more effective than either alone (210, 211).

Chimeric antigen receptor (CAR)-engineered NK cell therapies (CAR-NK cells) are in early clinical trials for solid and haematoligical malignancies (212). CAR-NK cells are NK cells modified *in vitro* to express a single-chain Fv fragment that targets a specific tumor antigen. These may prove a more attractive therapy than CAR-T cells, as there is less chance of them inducing cytokine storm or graft-versus-host disease, as it is not necessary to match HLA as strictly as for CAR-T cells (213).

The field of chimeric antigen receptor- (CAR) NK cell research also shows increasing promise, although clinical trials are still in the early stages. CAR-T cell therapies have so far been less effective at treating solid tumors than haematoligical (non-solid) malignancies, although whether this also true for CAR-NK cells is not clear (212, 213). CAR-NK cell infiltration into tumors is likely to be inhibited by the same features that affect general NK cell recruitment to tumors that we have discussed in this review; namely chemokine axes, extracellular matrix and soluble factors. These factors are likely also altered by radiotherapy, chemotherapy and immunotherapy (205).

## Discussion

Immunology is becoming more complicated as the appreciation of tissue complexity and cellular interactions grows. The phenotype, function, readiness to activate, retention and survival of immune cells are all dictated by the immediate environment. This is true for NK cell subsets, the study of which is catching up with T cells in that they too have a tissue-resident subset with distinct properties to those circulating (58). Furthermore, NK cells are influenced by contact with other cells, soluble mediators in the microenvironment and features of the extracellular matrix. Many new therapies look to promote NK cell activity in tumors; however, if these activated NK cells (e.g. by immune checkpoint inhibitors, or CAR-NK cell infusion) are unable to enter tumors, then these therapies may prove unsuccessful. A greater understanding of NK cell activity and the specific formation of the ECM following lung infection or in cancer needs to be assessed together.

The nomenclature surrounding tissue-resident NK cell subsets and other innate lymphoid cells requires further clarification as currently the disharmony makes it impossible to compare between studies, which we highlight in regard to the lung in **Table 1**. There may also be plasticity between NK cells and ILC subsets. Confusion also arises by looking at the NK cell population in bulk, rather than at least separating into CD16^−^ and CD16^+^ NK cells. It should be taken into consideration that CD16^−^ NK cells may be ‘ex-CD16^+^’ NK cells that have cleaved CD16 after activation (42, 43). An agreed phenotypic definition of the more differentiated CD16^+^/ex-CD16^+^ NK cell subset is needed, as is whether CD69 expression indicates tissue residency or merely activation.

It is not known where trNK cells fit in the differentiation process of CD16^−^ cells to CD16^+^ cells, or if they differentiate separately from traditional cNK cell subsets. However, TGFβ and/or IL-15 can induce the expression of CD103 and CD49a on blood-derived NK cells *in vitro*, (with CD16^−^ NK cells having a greater capacity to acquire this trNK cell-phenotype than CD16^+^ NK cells), indicating that perhaps these cells are not developmentally distinct but are instead reacting to specific signaling cues in the tissue environment (53, 58, 129, 153). Additionally, lung- and bone-marrow-derived cells with a trNK cell-like phenotype do not have increased capacity for self-renewal (unlike TRM cells), as measured by Ki-67 staining (21, 58, 218). As TGFβ is often elevated in the human TME, perhaps this is driving a switch to a trNK cell phenotype in lung tumors (2), as has been observed in mouse tumor models (157, 158).

The function of trNK cells remains a controversy as some studies have shown cells with this phenotype as tumor-promoting with low cytotoxicity (157–159), whereas others (including one study identifying these cells in human head and neck tumors) show they are highly cytotoxic with strong anti-tumor activity (53, 219). trNK cells in the liver display a form of immunological memory (20, 220), and a subset of trNK cells in the lung appear to also possess memory-like properties (21). CD8^+^ TRM cells express high amounts of the effector molecules ICOS and granzyme B; they also express high levels of inhibitory molecules CTLA4 and PD-1. As such, they function as rapid effector cells whose activity can be easily shut off (221). Given the similarity in gene expression and cell surface marker profile (58), trNK cells may have a similar function. However, an overall shared function of trNK cells throughout the body may not exist, as their function will likely depend on the specific tissue environment in which they reside; with dNK cells providing a clear example of this (55).

In the past NK cells were assigned a predominantly pro-inflammatory role. However, a regulatory role for NK cells is emerging that promotes inflammatory resolution (**Figure 2C**) (79, 100, 105, 106, 108–110). In a tumor setting, NK cells with an immunoregulatory phenotype could perhaps help drive tumor development. In mice, an immunoregulatory subset of NK cells expressing CD117 (c-Kit) and PD-L1 negatively regulate DC maturation, resulting in decreased DC priming of CD8^+^ T cells (222). When these NK cells were adoptively transferred into tumor-bearing mice, they promoted the development of lung metastases (222).

NK cell interactions with other immune cells is another area that warrants further study. As *in vitro* studies show, NK cells are an important helper cell to DCs, and NK cell–DC interactions have effects on both cell types (117, 194–198, 223). NK cells also interact with macrophages, but how these interactions may promote or inhibit tumor growth or affect the outcome of infection is difficult to study. Macrophages are one of the predominant immune cell types within lung tumors (124, 224, 225), so deciphering this cross-talk may help in the development of anti-tumor strategies that enable the ‘re-education’ of both NK cells and macrophages.

CAR-NK cell research offers the opportunity to alter many aspects of NK cell biology, such as enhancing chemokine receptor expression to aid NK cell recruitment, or modifying the repertoire of activating and inhibitory receptors to increase activity in tumors (226). Other NK-cell-based cancer therapies in development include anti-NKG2A monoclonal antibodies, recombinant IL-2 and recombinant IL-15 [reviewed by Bald et al. (74)]. However, studies with T cell-based immunotherapies show that targeting one immune cell type in the TME is often not enough to inhibit tumor growth. Combining chemotherapies, T cell-based and NK cell-based immunotherapies may be more effective and a number of combination clinical trials are currently in progress (74, 227).

In summary, NK cells are an important component of the immune response to infectious and non-infectious stimuli and many downstream immune pathways rely on their appropriate recruitment and activation. The field of tissue-specific training of innate immunity has exploded in recent years, such that it is impossible to understand fundamental responses of immune cells without taking into account their context. This is clearly also relevant to NK cells, which are influenced by immune and non-immune factors in the local environment, as well as by direct cell-to-cell contact. This communication is altered by the TME, resulting in low infiltration of NK cells into tumors. Promoting their recruitment into the TME, as well as their activation and retention, will be key for successful NK-cell based therapies. In addition, further research into the cross-talk between immune cells in the lung TME, such as that between NK cells and macrophages, may provide new areas for therapeutic intervention. The identification of trNK cells in the lung may also reveal novel roles for NK cells beyond their traditional cytotoxic activity; for example, in immune memory and tolerance.

## Author Contributions

MF wrote the first draft of the manuscript, EC and TH wrote sections of the manuscript. All authors contributed to revising, editing and approval of the submitted manuscript.

## Funding

This work was supported in part by grants from the Wellcome Trust (202865/Z/16/Z), and by Cancer Research UK *via* funding to the Cancer Research UK Manchester Centre (C147/A25254) and their Non-Clinical Training Programme.

## Conflict of Interest

The authors declare that the research was conducted in the absence of any commercial or financial relationships that could be construed as a potential conflict of interest.

## Publisher’s Note

All claims expressed in this article are solely those of the authors and do not necessarily represent those of their affiliated organizations, or those of the publisher, the editors and the reviewers. Any product that may be evaluated in this article, or claim that may be made by its manufacturer, is not guaranteed or endorsed by the publisher.
